# Identification of Novel Mutations in *ABCA4* Gene: Clinical and Genetic Analysis of Indian Patients with Stargardt Disease

**DOI:** 10.1155/2015/940864

**Published:** 2015-04-02

**Authors:** Rajani Battu, Anshuman Verma, Ramesh Hariharan, Shuba Krishna, Ravi Kiran, Jemima Jacob, Aparna Ganapathy, Vedam L. Ramprasad, Govindasamy Kumaramanickavel, Nallathambi Jeyabalan, Arkasubhra Ghosh

**Affiliations:** ^1^Department of Vitreoretina, Narayana Nethralaya, Bangalore, India; ^2^GROW Research Laboratory, Narayana Nethralaya, Narayana Health City, Bommasandra, Bangalore, Karnataka 560 099, India; ^3^Strand Life Sciences Pvt. Ltd., Bangalore, India; ^4^MedGenome Labs Pvt. Ltd., Bangalore, India; ^5^Singapore Eye Research Institute, Singapore

## Abstract

Stargardt disease (STGD) is the leading cause of juvenile macular degeneration associated with progressive central vision loss, photophobia, and colour vision abnormalities. In this study, we have described the clinical and genetic features of Stargardt patients from an Indian cohort. The next generation sequencing was carried out in five clinically confirmed unrelated patients and their family members using a gene panel comprising 184 retinal specific genes. Sequencing results were analyzed by read mapping and variant calling in genes of interest, followed by their verification and interpretation. Genetic analysis revealed* ABCA4 *mutations in all of the five unrelated patients. Among these, four patients were found with compound heterozygous mutations and another one had homozygous mutation. All the affected individuals showed signs and symptoms consistent with the disease phenotype. We report two novel* ABCA4 *mutations in Indian patients with STGD disease, which expands the existing spectrum of disease-causing variants and the understanding of phenotypic and genotypic correlations. Screening for causative mutations in patients with STGD using panel of targeted gene sequencing by NGS would be a cost effective tool, might be helpful in confirming the precise diagnosis, and contributes towards the genetic counselling of asymptomatic carriers and isolated patients.

## 1. Introduction

Stargardt disease (STGD: OMIM #248200/#600110) is an inherited genetic eye disease in which patients develop bilateral macular dystrophy leading to progressive loss of central vision in early childhood. It is the most common form of autosomal recessive juvenile macular dystrophy with a reported prevalence of 1 : 10000 [1, 2]. The disease is characterized by loss of central vision, fundus flavimaculatus, mottling or atrophy of the retinal pigment epithelium (RPE), bull's eye maculopathy, flecks in the macula, beaten-bronze macular appearance, and cone-rod dysfunction [3]. STGD is associated with accumulation of lipofuscin content in RPE cells, failure in removal of toxic substances, and significant photoreceptor cell death [4–6]. So far, mutations have been reported in six candidate genes (*ABCA4, ELOVL4, PROM1, PRPH2, *and* CRB1*) in various forms of panretinal dystrophies with the possible phenotype of STGD1 [7–12]. Mutations in* ABCA4 *gene are implicated in recessive STGD type I [13, 14].* ABCA4 *is a large gene which consists of 50 exons located in chromosome 1p13 [15]. It is a member of the subfamily A of the ATP-binding cassette (ABC) transporters that is expressed in the retinal outer segments of cone and rod photoreceptor cells. This gene is involved in the transport and clearance of all-transretinal aldehyde, a by-product of the retinoid cycle of vision, and other essential molecules across the disc membrane into the cytoplasm [16–18]. Defective ABCA4 leads to an accumulation of retinoids in the outer segment or the retinal pigment epithelium and nonhomogenous slowing of retinoid cycle kinetics, as observed in earlier studies in STGD patients [13]. In the animal model,* ABCA4*-knockout mice show deposition of lipofuscin-like substance in the RPE. Additionally, increased levels of lipofuscin components such as fluorescent diretinoid, A2E, and diretinal pyridinium are also observed [19, 20]. Interestingly, knockout of RDH8 (retinol dehydrogenase 8) which conducts all-transretinal aldehyde metabolism together with ABCA4 showed the retinal degeneration in mice [21]. Moreover, aged ABCA4 knockout albino mice display a mild retinal degeneration [22]. It is well known that* ABCA4* mutations are heterogeneous and cause various forms of retinal dystrophies, which include STGD, retinitis pigmentosa, age-related macular degeneration (AMD), and cone-rod dystrophy [12, 23–26]. In this study, we describe novel and reported* ABCA4* gene variants and associated phenotypes in Indian patients with STGD.

## 2. Methods

### 2.1. Study Subjects and Clinical Ascertainment

Five clinically confirmed unrelated patients with Stargardt disease and their available family members were enrolled for genetic analysis. Study subjects underwent a complete ophthalmic examination including measurement of visual acuity, intraocular pressure, slit lamp biomicroscopy, detailed fundus examination, fundus photography, fundus autofluorescence (FAF), spectral domain optical coherence tomography (SD-OCT), and full field electroretinography (ERG). Fifty normal controls without any history of eye diseases were included. This study adhered to the tenets of Declaration of Helsinki and was approved by the institutional ethical committee (IEC). Informed consent was obtained from all subjects for the study and publication of their data.

### 2.2. DNA Extraction

Five mL of peripheral blood samples was obtained from all subjects in EDTA coated vacutainers. Total genomic DNA was extracted using the salt precipitation method described previously [27]. Agarose gel electrophoresis was done to examine the integrity of genomic DNA.

### 2.3. Sample Preparation

Fifty nanograms of Qubit quantified DNA was used for library preparation using the Illumina Nextera protocol as per the manufacturer's instructions. This protocol uses transposon-based shearing of genomic DNA and allows the DNA to be “tagmented,” that is, fragmented and tagged simultaneously in the same tube. Limited cycle PCR was carried out to incorporate adaptors and sample specific barcodes to prepare the sample library. The tagged and amplified sample library was checked for quality using BioAnalyzer (Agilent, USA) and quantified.

### 2.4. Target Enrichment and Exome Sequencing

Approximately 500 nanograms of library was used for enrichment, involving two successive hybridization steps with target specific biotinylated probes. These probes were targeted at the exons of 184 genes with previously known pathogenic variations associated with multiple eye disorders. Bound DNA was pulled down using streptavidin beads. The target library was amplified using limited cycles of PCR. 6–10 pM of the enriched library was loaded for sequencing on to a MiSeq sequencer (Illumina, USA) using a standard v2 kit.

### 2.5. Data Analysis

*Alignment.* The trimmed fastq files were generated using MiSeq reporter from Illumina. The reads were aligned against the whole genome hg19 build using Strand NGS v1.6 (Strand Life Sciences) after trimming at least 1 bp at the 3′ end and any others with quality below 10. Reads which had length less than 25 bp after trimming were not considered for alignment. A maximum of 5 matches of alignment score of at least 90% were computed. The reads were realigned using the local realignment tool in Strand NGS. Reads that failed vendor QC, reads with average quality less than 20, reads with ambiguous characters, and translocated and single-mate flip reads were all filtered out.

### 2.6. Variant Calling

The Strand NGS variant caller was used to detect variants at locations in the target regions covered by a minimum of 10 reads with at least 2 variants reads. Variants with a decibel score of at least 50 were reported. Results were further compared with the dbSNP138 to identify novel variations. The potential deleterious effect of variants was determined by using various* in silico* prediction algorithms (PolyPhen, SIFT, Mutation Taster. PhyloP, LRT GERP++RS).

### 2.7. Variant Interpretation

Variants were then imported into Strandomics v1.0 (Strand Life Sciences) for annotation, prioritization, and reporting based on ACMG guidelines. Six known genes (*ABCA4, CNGB3, CRB1, ELOVL4, PROM1*, and* PRPH2*) for STGD were included for interpretation.

### 2.8. Validation of* ABCA4 *Mutations

The potential* ABCA4* variations were revalidated in all the affected individuals and in the available unaffected family members as well as 50 unrelated normal controls using polymerase chain reaction (PCR) and Sanger sequencing.* ABCA4 *coding exons (5, 17, 19, 35, 42, 46, and 47) and their flanking splice junctions were PCR amplified using the primers reported previously [28]. PCR amplicons were sequenced on a 3730xl DNA Sequencing Analyzer (Life Technologies). Sequencing results were analyzed in FinchTV software (Geospiza, Seattle, WA, USA) and compared with the reference databases (NM_000350, ENSEMBL, and ENST00000370225).

## 3. Results

**Figure 1:**
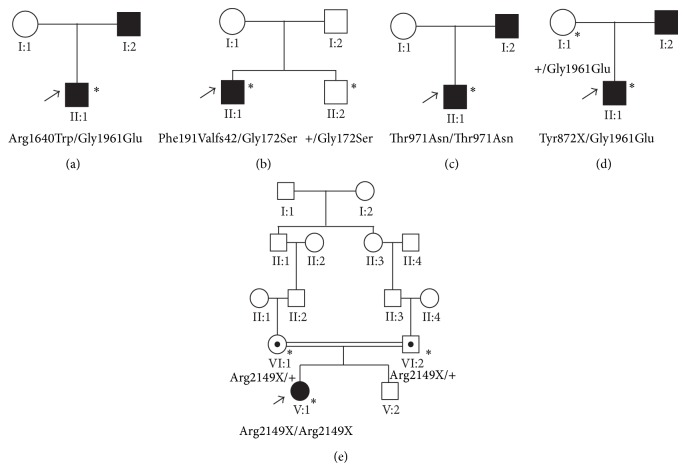
Pedigrees of five unrelated patients with Stargardt disease. (a) In Family SG-01 patient II:1 had a (p.Arg1640Trp) and (p.Gly1961Glu) compound heterozygous mutation. (b) In Family SG-02, the patient II:1 had novel (p.Phe191Valfs42) and reported p.Gly172Ser mutation. (c) In Family SG-03, the affected individual had a homozygous mutation (p.Thr971Asn). (d) Patient (II:1) from family SG-04_II:1 had a novel stop codon (p.Tyr872X) and (p.Gly1961Glu) mutation. (e) Patient (V:1) from a five-generation Indian family (SG-05) had a reported homozygous mutation (p.Arg2149X) in* ABCA4. *The asterisk denotes the individuals were included for the genetic analysis. Arrows indicate the proband in each family.

Genetic analysis was performed on a group of five unrelated patients with clinical findings of STGD (Figure 1). In this study, we performed NGS analysis in a panel of 184 genes encompassing multiple eye disorders including Stargardt disease. However the data analysis was performed for six known Stargardt related genes (*ABCA4*,* ELOVL4, CNGB3, PROM1, PRPH2, *and* CRB1*). This genetic analysis revealed a total of five mutations in the* ABCA4 *gene. One of these was a previously reported homozygous mutation (p.Arg2149X) with two asymptomatic heterozygous carriers in a five-generation consanguineous family. Two novel heterozygous mutations, p. Phe191Valfs42 and p.Tyr872X, were identified in two different patients along with previously reported heterozygous mutations (p.Gly172Ser, p.Gly1961Glu, resp.), resulting in a compound heterozygous state (p.Phe191Valfs42/p.Gly172Ser and p.Tyr872X/p.Gly1961Glu). In addition, previously reported compound heterozygous mutations (p.Arg1640Trp/p.Gly1961Glu) and a previously reported homozygous mutation (p.Thr971Asn) were found in two isolated cases. There are no pathogenic mutations that were identified in other candidate genes associated with STGD. The clinical features of the patients are described in Table 1. We also found nonpathogenic reported polymorphisms (SNPs) in the Stargardt related genes (*ABCA4*,* ELOVL4, CNGB3, PROM1, PRPH2, *and* CRB1*) which are listed in Table 2.

### 3.1. Family: SG-01

**Figure 2:**
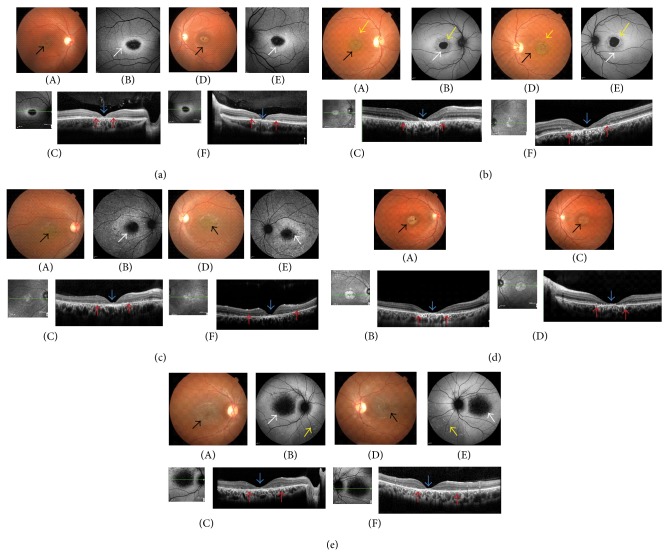
Clinical features of Stargardt patients with* ABCA4* mutations. (a) (SG-01_II:1): A and D show the fundus photos of the right and left eye. Note the atrophic macular lesions at the macula (*black arrows*). B and E show the corresponding fundus autofluorescence. Note the central area of hypoautofluorescence with a surrounding ring of hyperautofluorescence (*white arrows*). C and F show the foveal thinning (*blue arrows*) and the loss of photoreceptors and the external limiting membrane (*red arrows*). (b) (SG-02_II:1): A and D show the fundus photos of the right and left eye. Note the atrophic lesions at the macula (*black arrows*) and the flecks (*yellow arrows*). B and E show the corresponding fundus autofluorescence. Note the central area of hypoautofluorescence with a surrounding ring of hyperautofluorescence (*white arrows*). The* yellow arrow* corresponds to the flecks on FAF. C and F show the foveal thinning (*blue arrows*) and the loss of photoreceptors and the external limiting membrane (*red arrows*). (c) (SG-03_II:1): A and D show the fundus photo of the right and left eye. Note the central atrophic macula (*black arrows*) and the extensive flecks seen throughout the posterior pole. (B and E) FAF shows the corresponding area of central hypoautofluorescence (*white arrows*) surrounded by an area of hyperautofluorescence. Note the hyper- and hypoautofluorescence of flecks on FAF. (C and E) SD-OCT shows the central foveal thinning (*blue arrows*) and the loss of photoreceptors centrally (*red arrows*). (d) (SG-04_II:1): A and C show the fundus photos of the right and left eye. Note the atrophic lesions at the macula (*black arrows*). B and D show the foveal thinning (*blue arrows*) and the loss of photoreceptors and the external limiting membrane (*red arrows*). (e) (SG-04_V:1): A and D show the fundus photos of the right and left eye. Note the central atrophic macula (*black arrows*) and the extensive flecks seen throughout the posterior pole. (B and E) FAF shows the corresponding area of central hypoautofluorescence* (white arrows). *Note the hyperautofluorescence flecks (*yellow arrows*) that are not clear on the fundus pictures. (C and E) SD-OCT shows the central foveal thinning (*blue arrows*) and the extensive loss of photoreceptors (*red arrows*).

Patient (II:1) was a 27-year-old male from nonconsanguineous parents with progressive decrease of vision in both eyes (Figure 1(a)). His best corrected visual acuity was 6/60 and 6/36 in the right eye (RE) and left eye (LE), respectively. Anterior segment examination and the intraocular pressure (IOP) in both eyes were normal. Fundus showed an appearance of bull's eye maculopathy in both eyes. There were no visible flecks. Fundus autofluorescence (FAF) showed an area of hypoautofluorescence corresponding to the atrophic macula surrounded by a ring of hyperautofluorescence (Figure 2(a)). The ring of hyperautofluorescence corresponds to the lipofuscin-laden RPE cells while the central hypofluorescent area corresponds to an area where there is complete RPE atrophy. Full field electroretinography of both eyes was normal suggestive of group 1 STGD. SD-OCT of both eyes showed a central area of photoreceptor loss corresponding to the atrophy seen on the fundus and FAF (Figure 2(a)). Genetic analysis in patient (II:1) revealed two previously reported missense mutations, c.4918C>T (p.Arg1640Trp) and c.5882G>A (p.Gly1961Glu), in compound heterozygous state in the* ABCA4* gene. These compound heterozygous missense mutations were not present in 50 unrelated normal controls. The parents (I:1 and I:2) were clinically normal and did not consent to genetic analysis.

### 3.2. Family: SG-02

**Figure 3:**
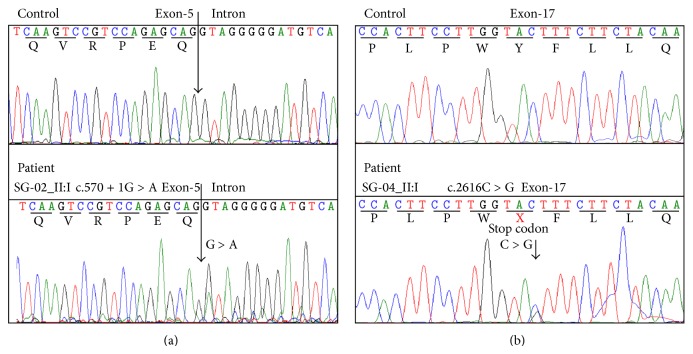
Sequencing analysis of two patients with novel* ABCA4* mutations. (a) and (b) show the comparison of sequence chromatograms of two patients (SG-02 II:1, SG-04_II:1) and the normal controls. Arrows indicate the positions of nucleotides change.

Patient (II:1) in the second family was a 31-year-old male with complaints of blurred vision for distance since the age of 20 years. He was the first sibling of nonconsanguineous parents (Figure 1(b)). His BCVA was 6/60, N24 in the RE and 4/60, N36 in the LE, respectively. Fundus examination of both eyes showed a central atrophic lesion with flecks restricted to the macula (Figure 2(b)). Anterior segment examination and the IOP were normal in both eyes. The central lesion was hypoautofluorescent on FAF corresponding to the area of RPE atrophy surrounded by hypo- and hyperfluorescent flecks. SD-OCT showed an area of photoreceptor loss corresponding to macular atrophy (Figure 2(b)). Full field ERG of both eyes was normal suggestive of group 1 STGD. Genetic analysis showed two mutations c.570+1G>A and c.514G>A (p.Gly172Ser) in compound heterozygote state. These mutations were not observed in 50 unrelated controls. The nucleotide change c.570+1G>A was a novel essential splice site mutation predicted to result in a frameshift at Phe 191. As a result, there would be a replacement of phenylalanine with valine followed by termination after 42 amino acids (p.Phe191Valfs42) (Figure 3(a)). The proband's parents were clinically normal and not tested for this mutation. The proband's brother (II:2) was normal on ophthalmic examination. Genetic screening revealed he was carrying only the p.Gly172Ser mutation but missing the essential splice site mutation c.570+1G>A.

### 3.3. Family: SG-03

The patient (II:1) in the third family was a 22-year-old male who presented with difficulties in night vision and blurred vision for distance since the age of 10. He was the only child of nonconsanguineous parents (Figure 1(c)). His BCVA was 6/60, N18 in the RE and 6/45, N18 in the LE, respectively. Slit lamp examination of the anterior segment revealed no abnormalities in both eyes with normal IOP. Fundus examination of both eyes showed an atrophic macula with retinal flecks (Figure 2(c)). FAF showed a central area of hypoautofluorescence corresponding to the atrophic macula and areas of multiple hyperautofluorescence corresponding to the flecks. SD-OCT of both eyes showed foveal thinning with extensive loss of photoreceptors (Figure 2(c)). The full field ERG of both eyes showed rod-cone dysfunction suggestive of group 3 STGD. Genetic analysis of patient (II:1) showed a previously reported homozygous mutation c.2912C>A (p.Thr971Asn) in* ABCA4* gene that was not present in 50 unrelated healthy controls analyzed. His parents were normal on ophthalmic examination. We were unable to examine the segregation of this mutation in the parents.

### 3.4. Family: SG-04

The patient (II:1) in the fourth family was a 26-year-old male with loss of central vision. He was the only child of nonconsanguineous parents (Figure 1(d)). His BCVA was 6/38, N12 in both eyes. Fundus of both eyes showed atrophic macular lesions (Figure 2(d)). Anterior segment examination and the IOP in both eyes were normal. There were no flecks; FAF showed central hypoautofluorescence corresponding to the central macular lesion. SD-OCT showed central loss of photoreceptors corresponding to the macular atrophy (Figure 2(d)). Full field ERG of both eyes was normal suggestive of group 1 disease. Genetic analysis of patient (II:1) revealed a novel mutation c.2616C>G (p.Tyr872X) and a previously reported mutation c.5882G>A (p.Gly1961Glu) occurring in a compound heterozygous state in the* ABCA4 *gene (Figure 3(b)). His unaffected mother (I:1) harbored only one heterozygous variant (p.Gly1961Glu) in* ABCA4*. His father (I:2) was clinically normal and was not included for genetic testing. These compound heterozygous mutations were not present in the 50 normal controls.

### 3.5. Family: SG-05

We identified a 12-year-old girl (V:1) from a five-generation Indian family with the characteristic features of STGD (Figure 1(e)). She presented with inability to see the letters on the classroom board since the age of 8 years. She was the first child of a consanguineous marriage. Her BCVA was 6/60, N12 and 6/38, N12 in the RE and LE, respectively, on the Snellen's chart. Slit lamp examination showed normal anterior segment and IOP in both eyes were normal. Fundus examination of both eyes showed mild temporal pallor of the optic nerves with an atrophic macula (Figure 2(e)). FAF of both eyes showed an area of hypoautofluorescence corresponding to the atrophic macula with multiple hyperautofluorescent areas corresponding to flecks. SD-OCT of both eyes showed foveal thinning with extensive photoreceptor loss. Full field ERG in both eyes showed a cone-rod dysfunction suggestive of group 2 STGD (Figure 2(e)). The parents and sibling were clinically normal. Genetic analysis of the patient (V:1) showed a previously reported homozygous change c.6445C>T (p.Arg2149X) in the* ABCA4* gene. Her unaffected parents (IV:1 and IV:2) were both heterozygous for the same mutation (p.Arg2149X) showing an asymptomatic carrier status. This mutation was not present in 50 unrelated controls.

## 4. Discussion

Mutational screening for Stargardt patients has not been described in detail in Indian patients earlier. An appreciation of the genetic basis is important for understanding the biology of the disease. In this study, we report five mutations in the* ABCA4* gene, two of which have not been previously reported, and we determine the clinical phenotype associated with these genotypes in five unrelated patients with STGD. Mutations in* ABCA4* gene are the most common causes of STGD in inherited as well as sporadic cases. Several pathogenic mutations in* ABCA4 *have been reported in various ethnic backgrounds of STGD and documented in the HGMD (http://www.biobase-international.com/product/hgmd) and dbSNP databases (http://www.ncbi.nlm.nih.gov/SNP/). Apart from STGD,* ABCA4 *mutations have also been reported in other ocular disorders including cone-rod dystrophy [29] and autosomal recessive retinitis pigmentosa [25].

The patient (SG-01_II:1) carries two pathogenic missense mutations p.Gly1961Glu and p.Arg1640Trp, both well-known disease-causing mutations in STGD. p.Gly1961Glu has been reported most frequently in Caucasian and Spanish patients with STGD [30, 31], in 11 probands, who exhibited compound heterozygosity with other missense variations in* ABCA4* [17]. p.Arg1640Trp was identified in a compound heterozygous Stargardt patient who harbored this variant along with p.Gly863Ala and p.Trp1408Arg.* In vitro* functional analysis showed a synergistic effect of the variants (p.Arg1640Trp and p.Trp1408Arg) in causing a severe reduction in ABCA4 protein expression [32]. The p.Gly1961Glu variation has been associated with a milder STGD phenotype; this is consistent with our findings that the patient had a normal full field ERG with no evidence of either cone or rod dysfunction. The fundus appearance in this patient was similar to the phenotype described previously in individuals carrying the variant p.Arg1640Trp [23].

Another compound heterozygous mutation (p.Phe191Valfs42/p.Gly172Ser) was found in the patient (SG-02_II:1) with typical signs of atrophic macula and paramacular flecks in both eyes. The missense amino acid substitution p.Gly172Ser was previously identified in STGD probands from South African and Italian cohorts [33, 34]. The novel essential splice site mutation c.570+1G>A (p.Phe191Valfs42) is predicted to cause missplicing of the gene, resulting in a frameshift and subsequent truncation in the protein. A neighboring mutation c.570G>C (p.Gln190His) has previously been reported in compound heterozygous state along with p.Gly1961Glu in a patient with STGD [14].

We found a homozygous missense mutation (p.Thr971Asn) in a patient (SG-03_II:1) with a severe phenotype of STGD (atrophic macula, extensive flecks, and rod-cone dysfunction on ERG). We could not check for p.Thr971Asn in the parents, who were possibly asymptomatic carriers. This missense change (p.Thr971Asn) has been previously observed in a STGD patient but not in any of the 96 healthy control individuals investigated in that study [35]. The mutation alters a residue within the Walker A ATP binding motif (residues 963–970), adjacent to the NBD1 domain, which is located in a highly conserved region. It has been reported that the NBD1 domain is responsible for basal ATPase activity [36]. Moreover, it has been shown that the variant p.Thr971Asn leads to reduced ATP-binding capacity and abolishes retinal-stimulated ATP hydrolysis [37].

Patient (SG-04_II:1) had a compound heterozygous change (p.Tyr872X, p.Gly1961Glu) and his unaffected mother harbored only one heterozygous variant (p.Gly1961Glu) in the* ABCA4* gene. It is well known that p.Gly1961Glu is found in compound heterozygous state along with another pathogenic variant in STGD [17]. The truncating mutation p.Tyr872X has not been previously reported. It is found in the topological domain of the ABCA4 chain and leads to premature truncation after 872 amino acids, which is likely to be nonfunctional and would be degraded or not be translated. Interestingly, nearby nonsense mutations in* ABCA4*, for example, p.Ser878X, have been identified in a Chinese patient with STGD [38].

Another homozygous mutation (p.Arg2149X) was identified in the young girl (SG-05_V:1) from a consanguineous family with a severe phenotype of STGD. Interestingly, a compound heterozygous combination of p.Arg2149X and another variant IVS45+1G>C has been found in patients who displayed an extensive atrophic appearance of the RPE and abnormal responses from rods and cones [39]. This mutation (p.Arg2149X) leads to a predicted truncated protein in ABC transporter 2 domain in ABCA4 and has been previously described in STGD patients from Japanese and Caucasian origins [40, 41]. There are very few reports describing the variants in* CNGB3, PROM1, PRPH2, ELOVL4,* and* CRB1* that are associated with rare causes of Stargardt or Stargardt-like disease phenotypes [8, 10–12]. Our results suggest that* ABCA4* pathogenic mutations might be a major cause in Indian patients with STGD; it has been reported in other ethnic groups as well [31, 33, 34, 40, 42, 43]. In this study we found the most frequent* ABCA4* mutation p.G1961E in two unrelated STGD patients; this has been reported in patients from African American and European ancestry with the frequencies of 2% and 11%, respectively [44, 45]. Recently, the different stages of an “optical gap” have been described on the SD-OCT in patients with p.G1961E mutations [46]. Stage 1 was characterized by mild disruption of the ellipsoid zone over the fovea, while stage 2 showed a progressive loss resulting in an apparent “optical gap” on the OCT. Stage 3 showed a collapse of this zone associated with RPE atrophy. In our study cohort, both the patients SG-01_II:1 and SG-04_II:1 who carried a p.G1961E did not demonstrate any optical gap phenotype on the SD-OCT (Figures 2(a) and 2(d)) and exhibited complete loss of photoreceptors and RPE atrophy in the foveal region.

Through our study, we showed the usefulness of next generation sequencing in making the precise molecular diagnosis of STGD and helping in clinical situation. However, a single candidate gene could not be selected for genetic analysis, since STGD is associated with variable clinical features and can be caused by six other known genes. The gold standard for identifying nucleotide changes is direct sequencing, but screening of genes like* ABCA4* (large and consists of 50 exons) or other STGD related genes is expensive but time consuming. The panel of targeted gene sequencing by NGS might be a cost effective tool, which enables the rapid sequencing of a large gene like* ABCA4* and multiple other genes simultaneously in large cohorts of patients with STGD. This could be a convenient tool for rapid determination of genotype-phenotype correlation in STGD or Stargardt-like diseases and this method could be routinely implemented in future gene therapy approaches in retinal dystrophies. Though we have suggested that performing clinical exome would give us an advantage of validating the clinical diagnosis, in our study cohort we identified nonambiguous STGD phenotype and had* ABCA4* gene mutation out of half a dozen other genes. However, this may not be the case when others use the same methodology in their clinics, where there could be clinical and genetic heterogeneity and in such circumstances clinical exome could come in handy for a clinician [47] in further management of the case for focused genetic counseling and also in helping parents to make appropriate reproductive choices.

In summary, we report here, for the first time, two novel* ABCA4* mutations from two unrelated STGD patients of Indian origin. Our genetic study on STGD patients has allowed better understanding of the significance of the mutational landscape in the* ABCA4* gene and may help in the genetic counseling of patients and asymptomatic carriers. The identified genetic variations and associated clinical phenotypes were consistent with other studies. We further confirm that screening of* ABCA4* mutations in patients with STGD is essential for clinical management and future therapeutic approaches.

## Figures and Tables

**Table 1:** Clinical features of STGD patients with *ABCA4* mutations.

Family ID	Age/sex	BCVA		Refraction		Fundus	FAF	SD-OCT	Full field ERG
		RE	LE	RE	LE				
SG-01_II:1	27/M	6/60, N8	6/36, N8	−2.5 DS	−2.75 DS	Macular atrophy, no flecks	Hypo-AF surrounded by ring of hyper-AF	IS/OS loss	Normal

SG-02_II:1	31/M	6/60, N34	4/60, N36	0	0	Macular atrophy, macular flecks	Hypo-AF surrounded by hyper- and hypo-AF flecks	IS/OS loss	Normal

SG-03_II:1	22/M	6/60, N18	6/45, N18	−1.25 DS/ −1.00 DC 90°	−1.50 DS/ −0.50 DC 20°	Macular atrophy, extensive flecks	Hypo-AF surrounded by hyper- and hypo-AF flecks	IS/OS loss	Rod-cone dysfunction

SG-04_II:1	26/M	6/38, N12	6/38, N12	−1.00 DS/ −0.5 DC 70°	−1.50 DS	Macular atrophy, no flecks	NA	IS/OS loss	Normal

SG-05_V:1	16/F	6/60, N12	6/38, N12	+0.5 DS/ −0.75 DC 5°	+0.5 DS/ −0.75 DC 170°	Macular atrophy, temporal pallor of optic discs	Hypo-AF surrounded by hyper-AF flecks	IS/OS loss	Cone-rod dysfunction

BCVA: best corrected visual acuity, RE: right eye, LE: left eye, BE: both eyes, IOP: intraocular pressure, PR: photoreceptors, AF: autofluorescent, SD-OCT: spectral domain optical coherence tomography, FAF: fundus autofluorescence, and ERG: electroretinography.

IS/OS: inner segment/outer segment layer, NA: not available.

**Table 2:** List of nonpathogenic variations identified in patients with STGD by NGS analysis.

Gene	Patient ID: SG-01_II:1		Patient ID: SG-02_II:1		Patient ID: SG-03_II:1		Patient ID: SG-04_II:1		Patient ID: SG-05_V:1	
	Variation identified^*^	SNP ID	Variation identified	SNP ID	Variation identified	SNP ID	Variation identified	SNP ID	Variation identified	SNP ID
*ABCA4 *	c.5844A>G	rs2275029	c.6006-16G>A	rs4147863	c.5844A>G	rs2275029	c.6006-16G>A	rs4147863	c.302+26A>G	rs2297634
	c.302+26A>G	rs2297634	c.302+26A>G	rs2297634	c.302+26A>G	rs2297634	c.1240-14C>T	rs4147830	c.-1086A>C	rs2151846
	c.1240-14C>T	rs4147830	c.5682G>C	rs1801574	c.5682G>C	rs1801574	c.1269C>T	rs4147831	c.-900A>T	rs3789452
	c.6729+21C>T	rs1800699	c.6729+21C>T	rs1800699	c.6285T>C	rs1801555	c.5682G>C	rs1801574	c.4774-17_4774-16delGT	rs199797077
	c.6285T>C	rs1801555	c.6285T>C	rs1801555	c.6006-16G>A	rs4147863	c.5715-25A>C	rs4147856		
	c.6006-16G>A	rs4147863	c.5715-25A>C	rs4147856	c.5715-25A>C	rs4147856	c.6729+21C>T	rs1800699		
	c.5603A>T	rs1801466	c.5844A>G	rs2275029	c.5836-11G>A	rs1800739	c.6285T>C	rs1801555		
	c.5836-11G>A	rs1800739	c.5836-11G>A	rs1800739	c.5814A>G	rs4147857	c.5814A>G	rs4147857		
	c.5814A>G	rs4147857	c.5814A>G	rs4147857	c.-1086A>C	rs2151846	c.5844A>G	rs2275029		
	c.5715-25A>C	rs4147856	c.2918+942C>T	rs3789398	c.-900A>T	rs3789452	c.5836-11G>A	rs1800739		
	c.5682G>C	rs1801574	c.-1086A>C	rs2151846						
	c.2918+942C>T	rs3789398	c.-900A>T	rs3789452						
	c.-1086A>C	rs2151846	c.5196+899C>T	rs145838948						
	c.4774-17_4774-16delGT	rs199797077								

*CRB1 *	c.964+475dupT	rs77569447	c.-149delA	rs369741574	c.-149delA	rs369741574	c.964+475dupT	rs77569447	c.964+475dupT	rs77569447
			c.964+475dupT	rs77569447	c.964+475dupT	rs77569447	c.3934-92G>T	rs1135810		
			c.2470-18A>G	rs7534863	c.2470-18A>G	rs7534863	c.2470-18A>G	rs7534863		
			c.3934-92G>T	rs1135810	c.3934-92G>T	rs1135810				

*CNGB3 *	c.2264A>G	rs3735972	c.494-11delT	rs36008065	c.-100T>A	rs62622781	c.494-11dupT	rs36008065	c.494-11dupT	rs36008065
	c.-580_-579delCA	rs138187783	c.-100T>A	rs62622781			c.-596G>T	rs10956955	c.-596G>T	rs10956955
	c.494-11delT	rs36008065								
	c.2214A>G	rs3735970								

*PROM1 *	c.2347-4dupC	rs34269395	c.2254-6C>G	rs3815344	c.2347-4dupC	rs34269395	c.303+6G>A	rs2078622	c.2347-6T>C	rs6449209
	c.2462+159dupT	rs3841512	c.2347-4dupC	rs34269395	c.759G>A	rs2286455			c.2486+3676G>C	rs3796859
	c.303+6G>A	rs2078622	c.2462+159dupT	rs3841512					c.2486+3681T>G	rs3796860
	c.1956+14G>A	rs4698436	c.303+6G>A	rs2078622						

*PRHP2 *	c.∗13C>T	rs361524			c.910C>G	rs361524	c.∗13C>T	rs361524	c.∗13C>T	rs361524

*ELOVL4 *	c.895A>G	rs3812153	c.-90G>C	rs62407622						
			c.800T>C	rs148594713	c.-90G>C	rs62407622	c.895A>G	rs3812153	c.-90G>C	rs62407622

^∗^Nomenclature based on c DNA position according to human genome variation society (HGVS).
